# Visual Object Tracking Based on Cross-Modality Gaussian-Bernoulli Deep Boltzmann Machines with RGB-D Sensors

**DOI:** 10.3390/s17010121

**Published:** 2017-01-10

**Authors:** Mingxin Jiang, Zhigeng Pan, Zhenzhou Tang

**Affiliations:** 1 Faculty of Computer and Software Engineering, Huaiyin Institute of Technology, Huai’an 223003, China; jmx@hyit.edu.cn; 2 Digital Media &Interaction Research Center, Hangzhou Normal University, Hangzhou 310012, China; zgpan@hznu.edu.cn; 3 College of Physics and Electronic Information Engineering, Wenzhou University, Wenzhou 325035, China

**Keywords:** Gaussian-Bernoulli deep Boltzmann machines, cross-modality features, Bayesian MAP, visual object tracking

## Abstract

Visual object tracking technology is one of the key issues in computer vision. In this paper, we propose a visual object tracking algorithm based on cross-modality featuredeep learning using Gaussian-Bernoulli deep Boltzmann machines (DBM) with RGB-D sensors. First, a cross-modality featurelearning network based on aGaussian-Bernoulli DBM is constructed, which can extract cross-modality features of the samples in RGB-D video data. Second, the cross-modality features of the samples are input into the logistic regression classifier, andthe observation likelihood model is established according to the confidence score of the classifier. Finally, the object tracking results over RGB-D data are obtained using aBayesian maximum a posteriori (MAP) probability estimation algorithm. The experimental results show that the proposed method has strong robustness to abnormal changes (e.g., occlusion, rotation, illumination change, etc.). The algorithm can steadily track multiple targets and has higher accuracy.

## 1. Introduction

Visual object tracking is one of the key research topics in the field of computer vision. In recent years, it has had a wide range of applications, such as robot navigation, intelligent video surveillance, andvideo measurement [1,2,3,4]. Despite many research efforts, visual object tracking is still regarded as a challenging problem due to changes in object appearance, occlusions, complex motion, illumination variation and background clutter [5].

A typical visual object tracking algorithm often includes three major components: a state transition model, an observation likelihood model and a search strategy. A state transition model is used to model the temporal consistency of the states of a moving object, whereas an observation likelihood model describes the object and observations based on visual representations. Undoubtedly, feature representation is the most important factor in visual object tracking. Most of existing RGB-D trackers [6,7,8] tend to use hand-crafted features to represent target objects, such as Harr-like features [9], histogram of oriented gradients (HOG) [10], and local binary patterns (LBP) [11]. Hand-crafted features aim to describe some pre-defined image patterns, but they cannot capture thecomplex and specific characteristics of target objects. Hand-crafted features may lead to the loss of unrecoverable information which is suitable for tracking in different scenarios.

With the rapiddevelopment of computation power and the emergence of large-scale visual data, deep learning has received much attention and had a promising performance in computer vision tasks, e.g., object tracking [12], object detection [13], and image classification [14]. Wang et al. proposed a so-called deep learning tracker (DLT) for robust visual tracking [15]. DLT trackers learn generic features from auxiliary natural images offline. ADLT tracker cannot obtain deep features with temporal invariance, which is important for visual object tracking. In [16], the authors proposed a video tracking algorithm using learned hierarchical features in which the hierarchical features are learned via a two-layer convolutional neural network. Ding et al. [17] proposed a new tracking–learning–data architecture to transfer a generic object tracker to a blur invariant object tracker without deblurring image sequences. One of the research focuses of this paper is how to use deep learning effectively to extract the features of the target objects in RGB-D data. 

To the best of our knowledge, the existing visual tracking methods using deep learning follow a similar procedure, which tracks objects in 2D sequences. Object tracking is performed over 2D video sequences in most early research works like TLD tracker [18], MIL tracker [19] and VTD tracker [20]. With the great popularity of affordable depth sensors, such as Kinect, Asus Xtion, and PrimeSense, an explosive growth of RGB-D data that can be used nowadays has been seen. Reliable depth images can provide valuable information to improve tracking performance. In [21], the author establishesa unified benchmark dataset of 100 RGB-D videos, which provide a foundation for further research in both RGB and RGB-D tracking. One of theresearch focuses of this paper is how to fuse RGB information and depth information effectively to improve the performance of visual object tracking in RGB-D data. 

To overcome the problems in the existing methods, we propose a visual object tracking algorithm based on cross-modality feature learning using Gaussian-Bernoulli deep Boltzmann machines (DBM) over RGB-D data. A cross-modality deep learning framework is usedto learn a robust tracker forRGB-D data. The cross-modality features of the samples are input into the logistic regression classifier, andthe observation likelihood model is established according to the confidence score of the classifier. We obtain the object tracking results over RGB-D data using aBayesian maximum a posteriori probability estimation algorithm. Experimental results show that such a cross-modality learning can improve the tracking performance.

The main contributions of this paper can be summarized as follows:

- We present a cross-modality Gaussian-Bernoulli deep Boltzmann machine (DBM) to learn the cross-modality features of target objects in RGB-D data. The proposed cross-modality Gaussian-Bernoulli DBM is constructed with two single-modality Gaussian-Bernoulli DBMs by adding an additional layer of binary hidden units on top of them, which can fuse RGB information and depth information effectively.
- A unified RGB-D tracking framework based on Bayesian MAP is proposed, in which the robust appearance description with cross-modality features deep learning, temporal continuity is fully considered in the state transition model.
- Extensive experiments are conducted to compare our tracker with several state-of-the-art methods on the recent benchmark dataset [21]. From experimental results, we can see that the proposed tracker performs favorably against the compared state-of-the-art trackers.

The remainder of the paper is organized as follows. First, feature learning over RGB-D data with cross-modality deep Boltzmann machines is described in the next section. Then we introduce our tracking framework in Section 3. The implementation of our proposed method is presented in Section 4. Experimental results and analysis are demonstrated in Section 5, and finally we draw conclusions in Section 6.

## 2. Related Work

### 2.1. Boltzmann Machine

**Figure 1:**
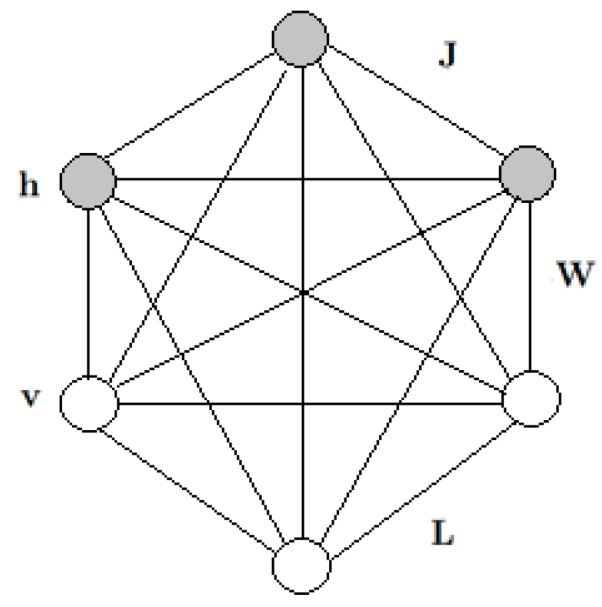
A general Boltzmann machine.

TheBoltzmann machine (BM) was proposed by Hinton and Sejnowski [22]. A Boltzmann machine is a feedback neural network consisting of fully connected coupled random neurons. The connections between neurons are symmetric, and there is no self-feedback. The outputs of neurons only have two states (active and inactive) which are expressed by 0 and 1, respectively. A set of visible units $v\in {\left\{0,1\right\}}^{D}$ and a set of hidden units $h\in {\left\{0,1\right\}}^{F}$ are included in BM (as shown in Figure 1). The visible units and hidden units are composed ofthe visible nodes and hidden nodes, and D and F represent the number of visible nodes and hidden layer nodes, respectively.

We formulate the energy function over the state {v,h} as:

(1) $$E(v,h;\Psi )=-v^{\prime }Wh-\frac{1}{2}h^{\prime }Rh-\frac{1}{2}v^{\prime }Lv-v^{\prime }B-h^{\prime }A$$

where Ψ={W,L,R,B,A} are the model parameters: W,L,R represent the symmetric interaction terms of visible nodes to hidden nodes, visible nodes to visible nodes, and hidden nodes to hidden nodes. The diagonal elements of L and R are set to 0. B and A are the threshold values of the visible layer and the hidden layer.

The model defines a probability distribution over a visible vector v as:

(2) $$P(v;\Psi )=\frac{P^{*}(v;\Psi )}{Z(\Psi )}=\frac{1}{Z(\Psi )}\sum_{h}exp(-E(v,h;\Psi ))$$

where $Z(\Psi )=\sum_{v}\sum_{h}exp(-E(v,h;\Psi ))$ is called the partition function, and $P^{*}$ is an unnormalized probability. 

The following formulations give the conditional distributions over hidden and visible units:

(3) $$P(h_{j}=1|v,h_{-j})=\sigma (\sum_{i=1}^{D}W_{ij}v_{i}+\sum_{m=1\backslash j}^{P}J_{jm}h_{j})$$

(4) $$P(v_{i}=1|h,v_{-i})=\sigma (\sum_{j=1}^{P}W_{ij}h_{j}+\sum_{k=1\backslash i}^{D}J_{ik}v_{i})$$

where σ(x)=1/(1+exp(−x)) is the logistic function.

### 2.2. Restricted Boltzmann Machine

**Figure 2:**
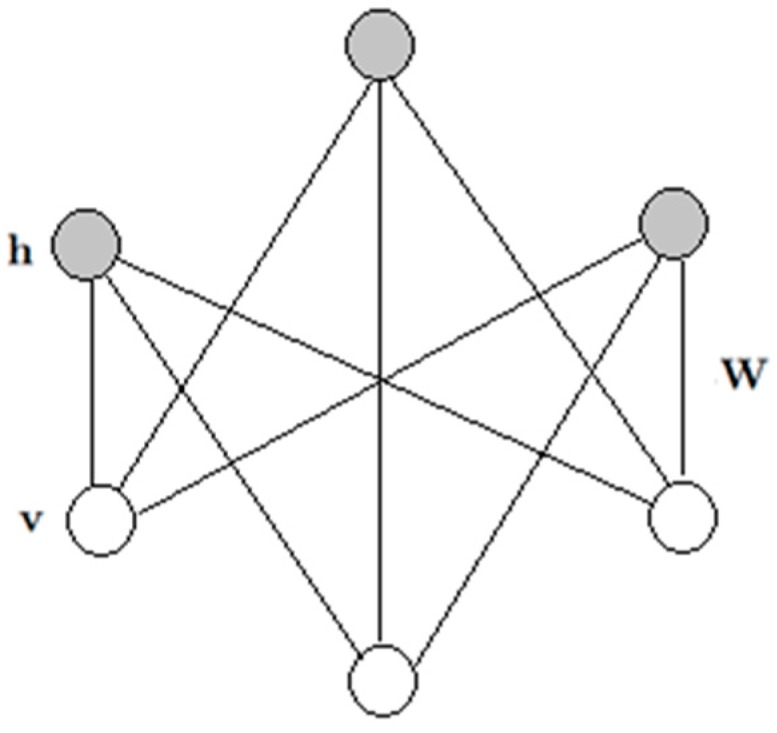
A restricted Boltzmann machine.

Setting both L=0 and R=0 in Equation (1), we will recover the model of a restricted Boltzmann machine (RBM), as shown in Figure 2.

A restricted Boltzmann machine(RBM) is a generative stochastic artificial neural networkthat can learn a probability distribution over its set of inputs. It is an undirected graphical model with each visible unit only connected to each hidden unit. The energy function over the visible and hidden units.

(5) $$E(v,h;\Psi )=-v^{\prime }Wh-v^{\prime }B-h^{\prime }A$$

where $E:{\left\{0,1\right\}}^{D+F}\to \mathbb{R}$, Ψ={W,A,B} are the model parameters. Equation (6) defines the joint probability distribution over the visible units $v\in {\left\{0,1\right\}}^{D}$ and hidden units $h\in {\left\{0,1\right\}}^{F}$.

(6) $$P(v,h;\Psi )=\frac{1}{Z(\Psi )}exp(-E(v,h;\Psi ))$$

where the normalizing factor Z(Ψ) denotes the partition function.

### 2.3. Gaussian-Bernoulli Restricted Boltzmann Machines

When inputs are real-valued images, we formulate the energy function of the Gaussian-Bernoulli RBM over the state {v,h} as follows [23]:

(7) $$E(v,h;\Psi )=-\sum_{i=1}^{D}\frac{{\left(v_{i}-b_{i}\right)}^{2}}{2\sigma_{i}^{2}}-\sum_{i=1}^{D}\sum_{j=1}^{F}W_{ij}h_{j}\frac{v_{i}}{\sigma_{i}}-\sum_{j=1}^{F}a_{j}h_{j}$$

where Ψ={a,b,W,σ} are the model parameters, $b_{i}$ and $a_{j}$ are biases corresponding to visible and hidden variables, respectively, $W_{ij}$ is the matrix of weights connecting visible and hidden nodes, and $\sigma_{i}$ is the standard deviation associated with a Gaussian visible variable $v_{i}$.

### 2.4. Gaussian-Bernoulli Deep Boltzmann Machine

**Figure 3:**
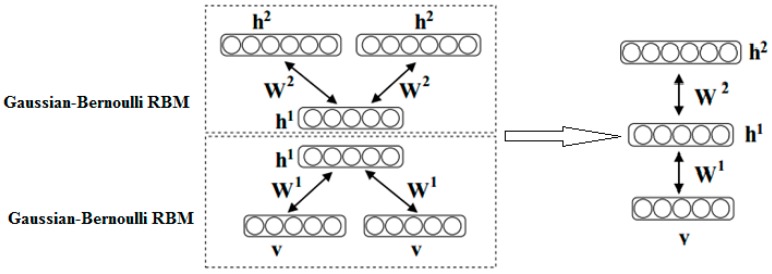
A Gaussian-Bernoulli Deep Boltzmann Machine.

A deep Boltzmann machine (DBM) [24] contains a set of visible units $v\in {\left\{0,1\right\}}^{D}$, and a sequence of layers of hidden units $h^{1}\in {\left\{0,1\right\}}^{L_{1}}$, $h^{2}\in {\left\{0,1\right\}}^{L_{2}}$, …, $h^{N}\in {\left\{0,1\right\}}^{L_{N}}$. Connections only exist between hidden units in adjacent layers. We illustrate a two-layer Gaussian-Bernoulli deep Boltzmann machine, consisting of learning a stack of modified Gaussian-Bernoulli RBMs (see Figure 3). 

The energy function of the joint configuration $\left\{v,h^{\left(1\right)},h^{\left(2\right)}\right\}$ is formulated as:

(8) $$E(v,h^{\left(1\right)},h^{\left(2\right)};\Psi )=-v^{\prime }W^{\left(1\right)}h^{\left(1\right)}-h^{{\left(1\right)}^{\prime }}W^{\left(2\right)}h^{\left(2\right)}$$

where $\Psi =\{W^{\left(1\right)},W^{\left(2\right)}\}$ are the model parameters, and $h=\{h^{\left(1\right)},h^{\left(2\right)}\}$ denote the set of hidden units. The probability distribution over a visible vector v can be modelled as:

(9) $$P(v;\Psi )=\frac{1}{Z(\Psi )}\sum_{h^{\left(1\right)},h^{\left(2\right)}}exp(-E(v,h^{\left(1\right)},h^{\left(2\right)};\Psi ))$$

## 3. Proposed Tracking framework

### 3.1. Feature Learning UsingCross-Modality Deep Boltzmann Machines over RGB-D Data

ABoltzmann machine (BM) is an effective tool in representing probability distribution over its inputs. Deep Boltzmann Machines (DBMs) have been successfully used in many application domains, e.g., topic modelling, classification, dimensionality reduction, feature learning, etc. According to the task, DBMs can be trained in either unsupervised or supervised ways. In this paper, we propose the cross-modality DBMs for feature learning in visual tracking over RGB-D data. In this section, we first describe how to establish cross-modality DBMs, review BMs, RBMs and Gaussian-Bernoulli restricted Boltzmann machines, then go over them in detail.

**Figure 4:**
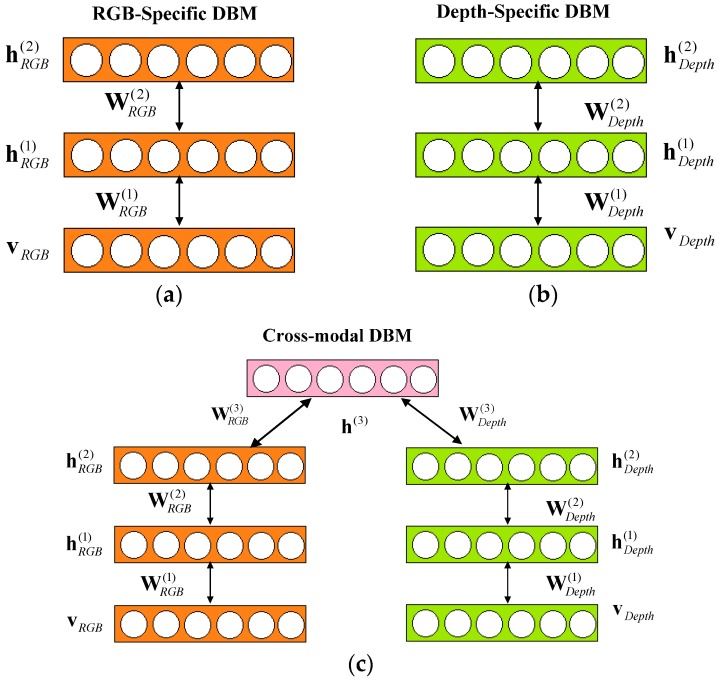
The illustration of the proposed cross-modal Gaussian-Bernoulli DBM. (**a**) RGB-specific two-layer Gaussian-Bernoulli DBM; (**b**) Depth-specific two-layer Gaussian-Bernoulli DBM; (**c**) a Cross-modal Gaussian-Bernoulli DBM.

Multimodal deep learning was proposed forvideo and audio [25,26]. In RGB-D data, we can also learn deep features over multiple modalities (RGB modality and depth modality). The proposed cross-modality DBM is constructed with two single-modality Gaussian-Bernoulli DBMs by adding an additional layer of binary hidden units on top of them (see Figure 4). Firstly, we model a RGB-specific Gaussian-Bernoulli DBM with two hidden layers as Figure 4a, where $v^{RGB}\in {\mathbb{R}}^{D}$ denotes a real-valued image input. Let $h^{\left(1RGB\right)}\in {\left\{0,1\right\}}^{F_{1}^{RGB}}$ and $h^{\left(2RGB\right)}\in {\left\{0,1\right\}}^{F_{2}^{RGB}}$ be the two layers of hidden units in the RGB-specific DBM. Then, the energy function of Gaussian-Bernoulli DBM over $\left\{v^{RGB},h^{RGB}\right\}$ is defined as:

(10) $$\begin{array}{r} E(v^{RGB},h^{\left(1RGB\right)},h^{\left(2RGB\right)};\Psi^{RGB})=\sum_{i=1}^{D}\frac{{\left(v_{i}^{\left(RGB\right)}-b_{i}^{\left(RGB\right)}\right)}^{2}}{2\sigma_{i}^{{\left(RGB\right)}^{2}}}-\sum_{i=1}^{D}\sum_{j=1}^{F_{1}^{RGB}}\frac{v_{i}^{\left(RGB\right)}}{\sigma_{i}^{\left(RGB\right)}}W_{ij}^{\left(1RGB\right)}h_{j}^{\left(1RGB\right)}\\ -\sum_{j=1}^{F_{1}^{RGB}}\sum_{l=1}^{F_{2}^{RGB}}W_{jl}^{\left(2RGB\right)}h_{j}^{\left(1RGB\right)}h_{l}^{\left(2RGB\right)}-\sum_{j=1}^{F_{1}^{RGB}}a_{j}^{\left(1RGB\right)}h_{j}^{\left(1RGB\right)}-\sum_{l=1}^{F_{2}^{RGB}}a_{l}^{\left(2RGB\right)}h_{l}^{\left(2RGB\right)} \end{array}$$

where $\sigma_{i}^{\left(RGB\right)}$ is thedeviation of the corresponding Gaussian model, and $\Psi^{RGB}$ is the parameter vector of RGB-specific Gaussian-Bernoulli DBM. Therefore, the joint distribution of the energy-based probabilistic model is defined through an energy function as:

(11) $$P(v^{RGB},h^{RGB};\Psi^{RGB})=\frac{1}{Z(\Psi^{RGB})}\sum_{h^{RGB}}exp(-E(v^{RGB},h^{RGB};\Psi^{RGB}))$$

where $Z(\Psi^{RGB})$ is the partition function. 

Similarly, the corresponding probability assigned to $v^{Depth}$ by Depth-specific DBM has the same form with Equation (11). Let $v^{Depth}\in {\mathbb{R}}^{K}$ denotes a real-valued depth image input. Let $h^{\left(1Depth\right)}\in {\left\{0,1\right\}}^{F_{1}^{Depth}}$ and $h^{\left(2Depth\right)}\in {\left\{0,1\right\}}^{F_{2}^{Depth}}$ be the two layers of hidden units in the Depth-specific DBM, as show in Figure 4b. The energy of the Gaussian-Bernoulli DBM and the joint distribution of the energy-based probabilistic model over $\left\{v^{Depth},h^{Depth}\right\}$ are defined as:

(12) $$\begin{array}{r} E(v^{Depth},h^{\left(1Depth\right)},h^{\left(2Depth\right)};\Psi^{Depth})=\sum_{i=1}^{D}\frac{{\left(v_{i}^{\left(Depth\right)}-b_{i}^{\left(Depth\right)}\right)}^{2}}{2\sigma_{i}^{{\left(Depth\right)}^{2}}}-\sum_{i=1}^{D}\sum_{j=1}^{F_{1}^{Depth}}\frac{v_{i}^{\left(Depth\right)}}{\sigma_{i}^{\left(Depth\right)}}W_{ij}^{\left(1Depth\right)}h_{j}^{\left(1Depth\right)}\\ -\sum_{j=1}^{F_{1}^{Depth}}\sum_{l=1}^{F_{2}^{Depth}}W_{jl}^{\left(2Depth\right)}h_{j}^{\left(1Depth\right)}h_{l}^{\left(2Depth\right)}-\sum_{j=1}^{F_{1}^{Depth}}a_{j}^{\left(1Depth\right)}h_{j}^{\left(1Depth\right)}-\sum_{l=1}^{F_{2}^{Depth}}a_{l}^{\left(2Depth\right)}h_{l}^{\left(2Depth\right)} \end{array}$$

(13) $$P(v^{Depth},h^{\left(1Depth\right)},h^{\left(2Depth\right)};\Psi^{Depth})=\frac{1}{Z(\Psi^{Depth})}\sum_{h^{\left(1Depth)(2Depth\right)}}exp(-E(v^{Depth},h^{\left(1Depth\right)},h^{\left(2Depth\right)};\Psi^{Depth}))$$

where $\sigma_{i}^{\left(Depth\right)}$ is deviation of the corresponding Gaussian model, and $\Psi^{Depth}$ is the parameter vector of Depth-specific Gaussian-Bernoulli DBM.

Let $v^{RGB}\in {\mathbb{R}}^{D}$ and $v^{Depth}\in {\mathbb{R}}^{K}$ denote a real-valued RGB input and a real-valued depth input respectively. Consider modeling an image-depth DBM with three hidden layers, let $\left\{v^{RGB},v^{Depth}\right\}$ be real-valued Gaussian variables, and $\left\{h^{\left(1RGB\right)},h^{\left(2RGB\right)},h^{\left(1Depth\right)},h^{\left(2Depth\right)},h^{\left(3\right)}\right\}$ be binary stochastic hidden units. Let $h^{\left(1RGB\right)}\in {\left\{0,1\right\}}^{F_{1}^{RGB}}$ and $h^{\left(2RGB\right)}\in {\left\{0,1\right\}}^{F_{2}^{RGB}}$ be the two layers of hidden units in the RGB-specific two layer DBM. Similarly, let $h^{\left(1Depth\right)}\in {\left\{0,1\right\}}^{F_{1}^{Depth}}$ and $h^{\left(2Depth\right)}\in {\left\{0,1\right\}}^{F_{2}^{Depth}}$ be the two layers of hidden units in the depth-specific two layer DBM. The energy of the proposed cross-modality Gaussian-Bernoulli DBM over {v,h} can be defined as:

(14) $$\begin{array}{l} E(v,h;\Psi^{cross-modality})=\sum_{i=1}^{D}\frac{{\left(v_{i}^{\left(RGB\right)}-b_{i}^{\left(RGB\right)}\right)}^{2}}{2\sigma_{i}^{{\left(RGB\right)}^{2}}}-\sum_{i=1}^{D}\sum_{j=1}^{F_{1}^{RGB}}\frac{v_{i}^{\left(RGB\right)}}{\sigma_{i}^{\left(RGB\right)}}W_{ij}^{\left(1RGB\right)}h_{j}^{\left(1RGB\right)}\\ -\sum_{j=1}^{F_{1}^{RGB}}\sum_{l=1}^{F_{2}^{RGB}}h_{j}^{\left(1RGB\right)}W_{jl}^{\left(2RGB\right)}h_{l}^{\left(2RGB\right)}-\sum_{l=1}^{F_{2}^{RGB}}\sum_{p=1}^{F_{3}^{RGB}}h_{j}^{\left(2RGB\right)}W_{lp}^{\left(3RGB\right)}h_{p}^{\left(3RGB\right)}-\sum_{j=1}^{F_{1}^{RGB}}a_{j}^{\left(1RGB\right)}h_{j}^{\left(1RGB\right)}\\ -\sum_{l=1}^{F_{2}^{RGB}}a_{l}^{\left(2RGB\right)}h_{l}^{\left(2RGB\right)}+\sum_{i=1}^{K}\frac{{\left(v_{i}^{\left(Depth\right)}-b_{i}^{\left(Depth\right)}\right)}^{2}}{2\sigma_{i}^{{\left(\mathrm{Depth}\right)}^{2}}}-\sum_{i=1}^{K}\sum_{j=1}^{F_{1}^{Depth}}\frac{v_{i}^{\left(Depth\right)}}{s_{i}^{\left(Depth\right)}}W_{ij}^{\left(1Depth\right)}h_{j}^{\left(1Depth\right)}\\ -\sum_{j=1}^{F_{1}^{Depth}}\sum_{l=1}^{F_{2}^{Depth}}h_{j}^{\left(1Depth\right)}W_{jl}^{\left(2Depth\right)}h_{l}^{\left(2Depth\right)}-\sum_{l=1}^{F_{2}^{Depth}}\sum_{p=1}^{F_{3}^{Depth}}h_{j}^{\left(2Depth\right)}W_{lp}^{\left(3Depth\right)}h_{p}^{\left(3Depth\right)}-\sum_{j=1}^{F_{1}^{Depth}}a_{j}^{\left(1Depth\right)}h_{j}^{\left(1Depth\right)}\\ -\sum_{l=1}^{F_{2}^{Depth}}a_{l}^{\left(2Depth\right)}h_{l}^{\left(2Depth\right)}-\sum_{p=1}^{F_{3}}a_{p}^{\left(3\right)}h_{p}^{\left(3\right)} \end{array}$$

Therefore, the joint probability distribution over the cross-modal input $\left\{v^{RGB},v^{Depth}\right\}$ can be written as:

(15) $$\begin{array}{l} P(v^{RGB},v^{Depth};\Psi^{cross-modality})=\sum_{h^{\left(2RGB\right)},h^{\left(2Depth\right)},h^{\left(3\right)}}P(h^{\left(2RGB\right)},h^{\left(2Depth\right)},h^{\left(3\right)})(\sum_{h^{\left(1RGB\right)}}P(v^{RGB},h^{\left(1RGB\right)},h^{\left(2RGB\right)})\\ (\sum_{h^{\left(1Depth\right)}}P(v^{Depth},h^{\left(1Depth\right)},h^{\left(2Depth\right)})\\ =\frac{1}{Z(\Psi^{cross-modality})}\sum_{h}exp(-\sum_{i}\frac{{\left(v_{i}^{RGB}\right)}^{2}}{2s_{i}^{2}}+\sum_{ij}\frac{v_{i}^{\left(RGB\right)}}{s_{i}}W_{ij}^{\left(1RGB\right)}h_{j}^{\left(1RGB\right)}+\sum_{jl}W_{jl}^{\left(2RGB\right)}h_{j}^{\left(1RGB\right)}h_{l}^{\left(2RGB\right)}\\ -\sum_{i}\frac{{\left(v_{i}^{Depth}\right)}^{2}}{2s_{i}^{2}}+\sum_{ij}\frac{v_{i}^{\left(Depth\right)}}{s_{i}}W_{ij}^{\left(1Depth\right)}h_{j}^{\left(1Depth\right)}+\sum_{jl}W_{jl}^{\left(2Depth\right)}h_{j}^{\left(1Depth\right)}h_{l}^{\left(2Depth\right)}\\ +\sum_{lp}W^{\left(3RGB\right)}h_{l}^{\left(2RGB\right)}h_{p}^{\left(3\right)}+\sum_{lp}W^{\left(3Depth\right)}h_{l}^{\left(2Depth)\right)}h_{p}^{\left(3\right)}) \end{array}$$

where $\Psi^{cross-modality}$ is the parameter vector of cross-modality Gaussian-Bernoulli DBM. The task of learning the cross-modality Gaussian-Bernoulli DBM is the maximum likelihood learning for Equation (6) with respect to the model parameters.

### 3.2. Bayesian Framework

In this paper, the object tracking is formulated as a hidden state variable Bayesian maximum a posteriori (MAP) estimation problem in the Hidden Markov model. Given a set of observed variables $Z_{t}=\{Z_{1},Z_{2},\ldots ,Z_{t}\}$, we can estimate the hidden state variable $X_{t}=\left\{X_{t}^{1},X_{t}^{2},\ldots \ldots X_{t}^{N}\right\}$ by using Bayesian MAP theory [27].

The posteriori probability distribution according to the Bayesian theory can be modelled as the following derivation:

(16) $$p(X_{t}|Z_{t})\propto p(Z_{t}|X_{t})\int p(X_{t}|X_{t-1})p(X_{t-1}|Z_{t-1})dX_{t-1}$$

where $p(Z{}_{t}|X_{t})$ stands for an observation likelihood model and $p(X_{t}|X_{t-1})$ is called a state transition model for two consecutive frames. We can obtain the optimal state ${\hat{X}}_{t}$ among all the candidates through maximum posterior probability estimation:

(17) $${\hat{X}}_{t}=\underset{X_{t}}{\arg\max}\;p(X_{t}|Z_{t})$$

#### 3.2.1. State Transition Model 

The state variable is defined as $X_{t}=\{x_{t},y_{t},\theta_{t},s_{t},\alpha_{t},\phi_{t}\},$ which includes the six parameters of the motion affine transformation, where $x_{t}$ and $y_{t}$ denote the *x*-direction and *y*-direction translation of the object in the frame *t* respectively, $\theta_{t}$ represents the rotation angle, $s_{t}$ stands for the scale change, $\alpha_{t}$ denotes the aspect ratio, and $\phi_{t}$ represents skew direction at time *t*.

We assume that the candidate states are generated according to Gaussian distribution:

(18) $$p(X_{t}|X_{t-1})\;=\;N(X_{t};X_{t-1},\Sigma )$$

where Σ is a diagonal covariance matrix whose diagonal elements are $\sigma_{x}^{2},\sigma_{y}^{2},\sigma_{\theta }^{2},\sigma_{S}^{2},\sigma_{\alpha }^{2},\sigma_{\phi }^{2}.$

#### 3.2.2. Observation Likelihood Model

In this paper, the observation model that we use is discriminative. A binary linear classifier is adopted to classify tracking observations into object class and background class during tracking. Observations are represented using features learned from the DBM introduced previously. We can obtain a training dataset with approximate labels after extracting features of positive and negative samples. Deep representations are likely to be linearly separable, and linear classifiers are less prone to overfitting. We adopt the logistic regression classifier owing to its capability of providing predictions in probability estimation. 

Let $h_{i}^{3}\in {\mathbb{R}}^{r\times 1}$ denote the deep feature for the i-th training sample, and $y_{i}\in \{-1,+1\}$ represent the label for the i-th training sample. $Z^{+}=[h_{1^{+}}^{3},h_{2^{+}}^{3},\ldots ,h_{D^{+}}^{3}]\in {\mathbb{R}}^{r\times D^{+}}$ stands for the positive training set with their respective labels as $Y^{+}=[y_{1^{+}},y_{2^{+}},\ldots ,y_{D^{+}}]\in {\left\{-1,+1\right\}}^{D^{+}\times 1}$. Similarly, $Z^{-}=[h_{1^{-}}^{3},h_{2^{-}}^{3},\ldots ,h_{D^{-}}^{3}]\in {\mathbb{R}}^{r\times D^{-}}$ represents the negative training set with their respective labels as $Y^{-}=[y_{1^{-}},y_{2^{-}},...,y_{D^{-}}]\in {\left\{-1,+1\right\}}^{D^{-}\times 1}$. Training the logistic regression classifier by optimizing:

(19) $$\underset{\pm w}{\min}\;C^{+}\sum_{i^{+}=1}^{D^{+}}\log(1+e^{y_{i^{+}}\pm w^{T}\pm h_{i^{+}}^{\left(3\right)}})+C^{-}\sum_{i^{-}=1}^{D^{-}}\log(1+e^{y_{i^{-}}\pm w^{T}\pm h_{i^{-}}^{\left(3\right)}})$$

where $C^{+}\in \mathbb{R}$ is the parameter to weight the logistic cost of the positive-class and $C^{-}\in \mathbb{R}$ is the parameter to weight the logistic cost of the negative-class logistic. Weight regularization w is added to the cost function in Equation (19) to reduce overfitting. In the prediction stage, the confidence score of the trained logistic regression classifier can be computed as follows:

(20) $$p(Z_{t}|X_{t})=\frac{1}{1+e^{-(\pm w^{T}\pm z_{t})}}$$

## 4. The Implementation of Our Proposed Method

**Figure 5:**
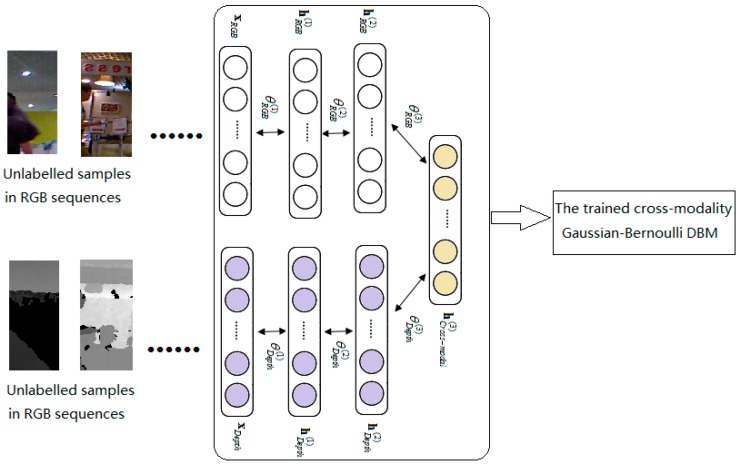
Offline learning of the proposed cross-modality Gaussian-Bernoulli DBM.

**Figure 6:**
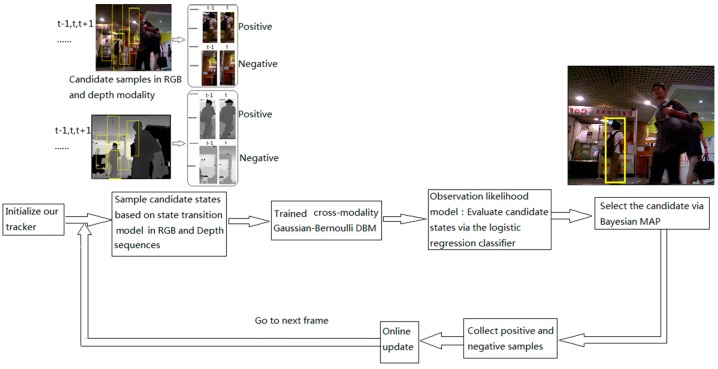
The process of object tracking online based on Bayesian MAP.

Our method has two major components, which are shown in Figure 5 and Figure 6. In the first place, as demonstrated in Figure 5, unlabeled patches in RGB and depth modality are used to train the cross-modality Gaussian-Bernoulli DBM offline.

Then, the trained cross-modality Gaussian-Bernoulli DBM is transferred to an observational model for visual tracking online based on Bayesian MAP, as shown in Figure 6.

## 5. Experimental Results and Analysis

The experiments of our proposed tracking algorithm is implemented on MATLAB R2014a, Intel(R) Core(TM) i7-4712MQ, CPU@3.40 GHz and TITAN GPU, 8.00 GB RAM, Windows 8.1 operating system, in Beijing, China. 

### 5.1. Qualitative Evaluation

In order to show the robustness of the visual object tracking algorithm discussed in this paper, we compare our tracker with several state-of-the-art methods on arecent benchmark dataset [21] in different environments with heavy or long-time partial occlusion, rotation, scale change, and fast motion. Given the limited space, in this section we only list four of them to show the experimental results and the forms of data statistics. 

We compare our method with several state-of-the-art trackers, including TLD Tracker[18], MIL Tracker [19],VTD Tracker [20], and RGB-D Tracker [28], CT Tracker [29], Struck Tracker [30], Deep Tracker [15], and Multi-cues Tracker [31],andwe ranthe experiments based on the code provided by the authors. 

**Figure 7:**
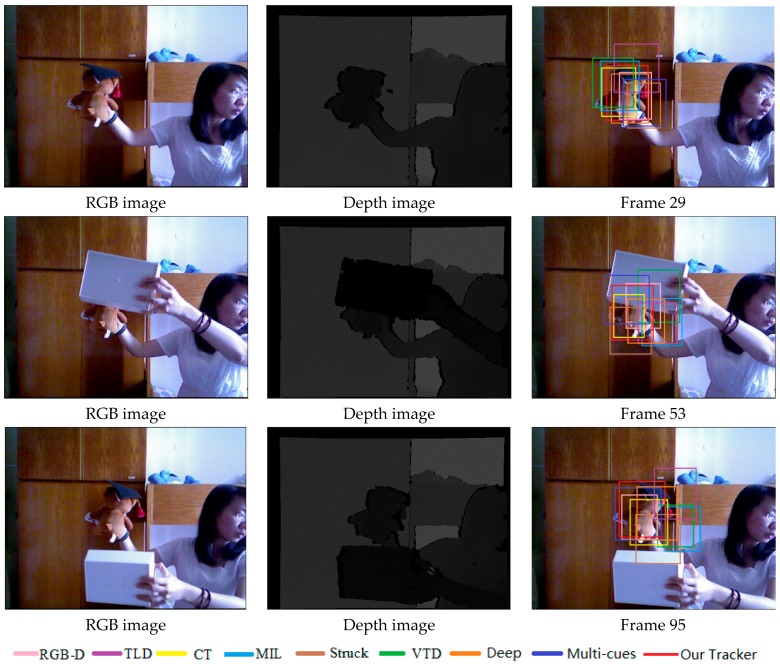
The tracking results on the test video 1 obtained by different methods.

Figure 7 demonstrates that our method performs well in terms of rotation, scale and position when the object undergoes severe occlusion. The MIL tracker and VTD tracker are sensitive to occlusion.

**Figure 8:**
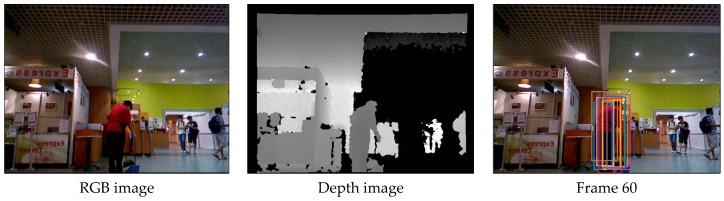

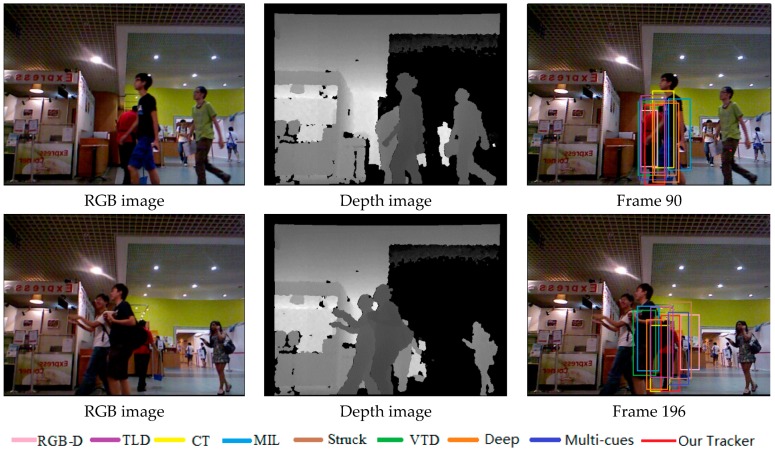
The tracking results on the test video 2 obtained by different methods.

Figure 8 shows the tracking results in the sequence with long-time partial occlusion, pose change and background clutter. We can see that the RGBD, MIL and VTD methods do not perform well and they are less effective in this case.

**Figure 9:**
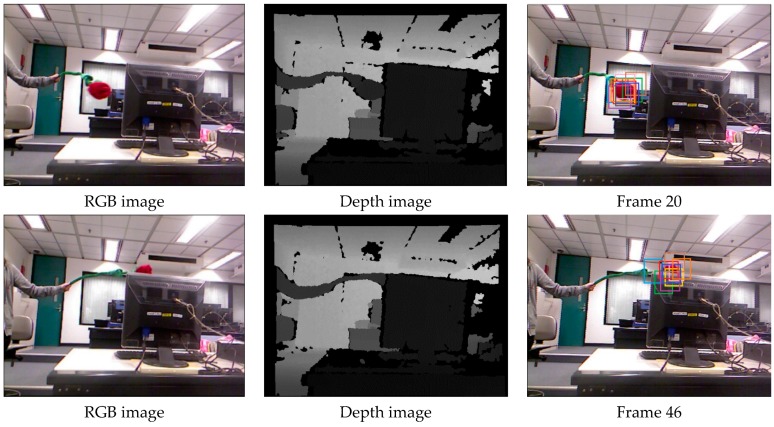

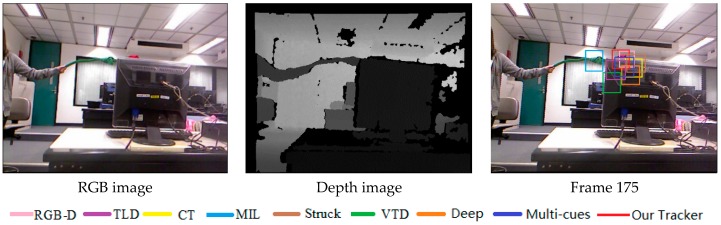
The tracking results on the test video 3 obtained by different methods.

Figure 9 illustrates the tracking results on the test video with severe occlusion, appearance change and fast motion. From the results, we can notice that the TLD, MIL and VTD methods are sensitive to target appearance change or occlusion.

**Figure 10:**
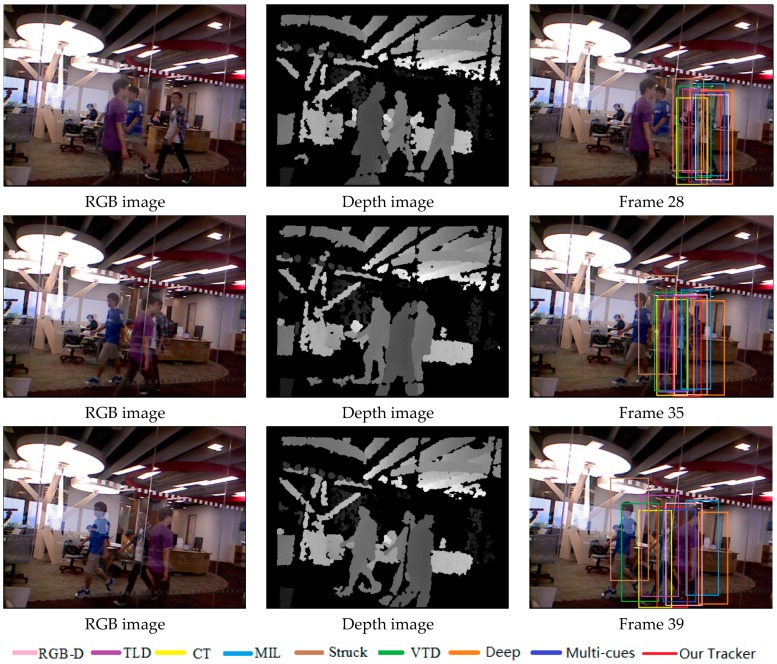
The tracking results on the test video 4 obtained by different methods.

Figure 10 shows the tracking results in the sequence with all occlusion, pose change and background clutter. We can see that the Stuck, MIL and VTD methods do not perform well and they are less effective in this case.

**Figure 11:**
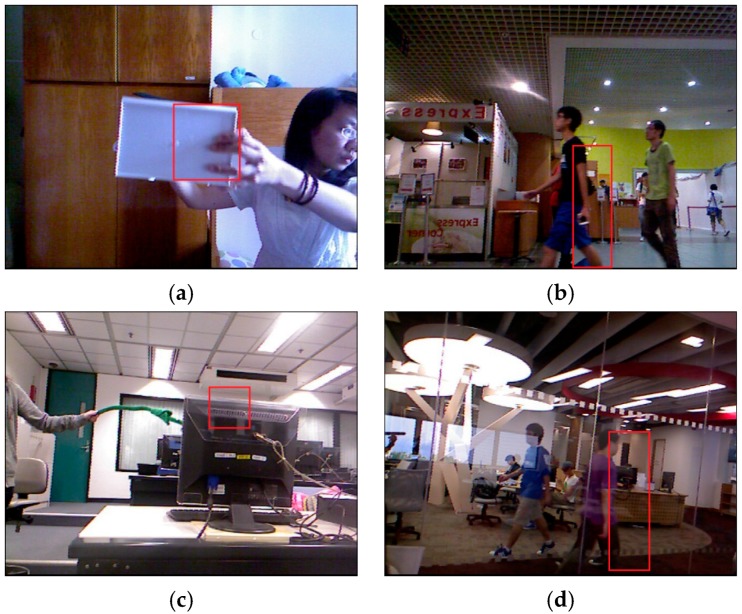
The“bad” tracking results of our method. (**a**) Frame 47 in test video 1; (**b**) Frame 93 in test video 1; (**c**) Frame 66 in test video 3; (**d**) Frame 37 in test video 3.

Figure 11 illustrates the “bad” tracking results of our method,meaning frames where tracking failures are observed. When the objects are all occluded, the tracking results of our method experience a drift phenomenon.

As shown in experimental results, the proposed tracking method performs favorably against the state-of-the-art tracking methods in handling challenging video sequences, but there are some limitations for our method. The robustness of the proposed tracking method is not strong enough to solve allocclusion and abrupt movement.

### 5.2. Quantitative Evaluation

We use two measurements to quantitatively evaluate tracking performances. The first one is called average center location error [32] which measures distances of centers between tracking results and ground truths in pixels. The second one is called success rate (SR) which is calculated according to $\frac{area(R_{T}\cap R_{G})}{area(R_{T}\cup R_{G})}$ and indicates theextent of region overlapping between tracking results $R_{T}$ and $R_{G}$.

**Figure 12:**
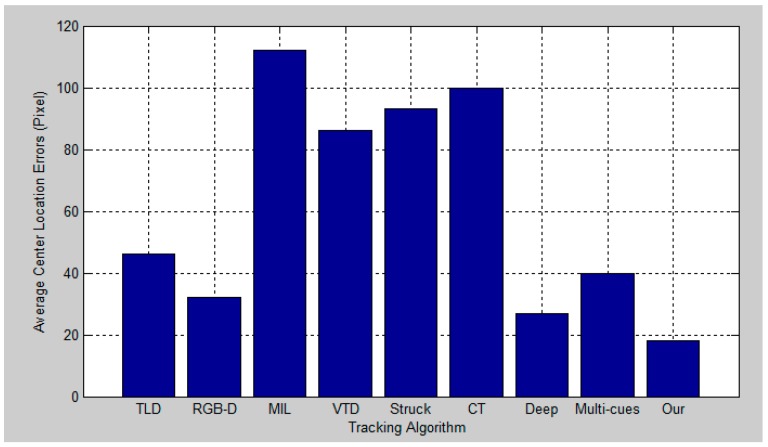
Quantitative evaluation in terms of average center location error (in pixel) for the first experiment.

**Figure 13:**
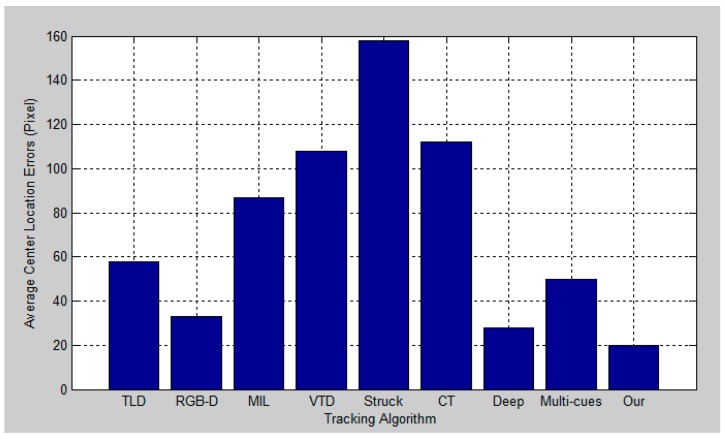
Quantitative evaluation in terms of average center location error (in pixel) for the second experiment.

**Figure 14:**
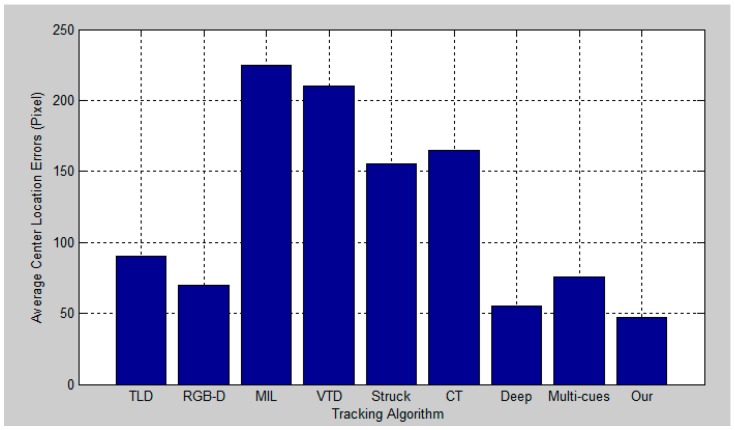
Quantitative evaluation in terms of average center location error (in pixel) for the third experiment.

**Figure 15:**
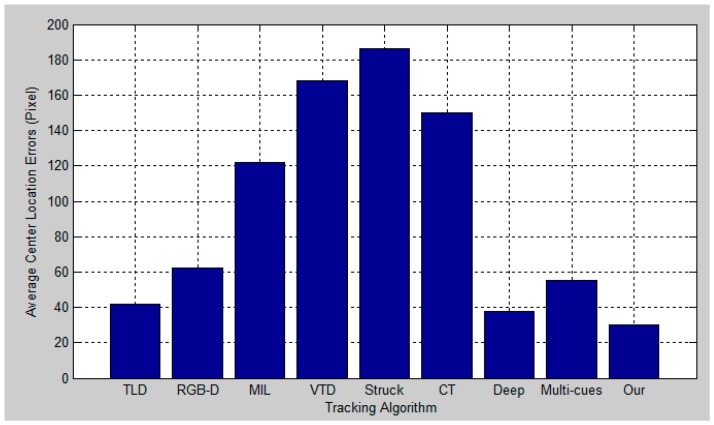
Quantitative evaluation in terms of average center location error (in pixel) for the fourth experiment.

Figure 12, Figure 13, Figure 14 and Figure 15 report the average center location errors of different tracking methods over three test videos. The comparison results show that the proposed method has a smaller average center location error than the state-of-the-art methods indifferent situations. 

Table 1 reports the success rates, where larger scores mean more accurate results. 

Table 2 lists the average speed of each method on the recent benchmark dataset [21]. The average speed of our method is 0.14 fps, implemented in Matlab without optimization for speed. The fine-tuning of our method is time-consuming.

## 6. Conclusions

By analyzing the problems of the existing technologies, this paper proposes a visual object tracking algorithm based on cross-modality features learning using Gaussian-Bernoulli deep Boltzmann machines (DBM) over RGB-D data. We extract cross-modality features of the samples in RGB-D video data based on across-modality Gaussian-Bernoulli DBM and obtain the object tracking results over RGB-D data using aBayesian maximum a posteriori probability estimation algorithm. The experimental results show that the proposed method greatly improves the robustness and accuracy of thealgorithm. In the future, we will extend the proposed method to solve other vision problems (e.g., object detection, face recognition, etc.).

## Figures and Tables

**Table 1:** The evaluation results of SR under different categorizations.

Method	Object Type		Movement		Occlusion	
	Human	Animal	Fast	Slow	Yes	No
Our Tracker	80.1%	72.9%	77.5%	82.3%	81.2%	82.6%
TLD Tracker	29.0%	35.1%	29.7%	51.6%	33.8%	38.7%
VTD Tracker	30.9%	48.8%	37.2%	57.3%	28.3%	63.1%
MIL Tracker	32.2%	37.2%	31.5%	45.5%	25.6%	49.0%
RGB-D Tracker	47.1%	47.0%	51.8%	56.7%	46.9%	61.9%
Struck Tracker	35.4%	47.0%	39.0%	58.0%	30.4%	63.5%
CT Tracker	31.1%	46.7%	31.5%	48.6%	34.8%	46.8%
Deep Tracker	72.1%	64.8%	70.1%	76.3%	71.4%	72.6%
Multi-cues Tracker	33.2%	49.5%	52.3%	55.6%	44.7%	57.5%

**Table 2:** The average speed of each method on the recent benchmark dataset [21].

Method	The Average Speed (fps)
Our Tracker	0.14
TLD Tracker	28.5
VTD Tracker	6.7
MIL Tracker	38.9
RGB-D Tracker	2.6
Struck Tracker	20.8
CT Tracker	64.7
Deep Tracker	0.23
Multi-cues Tracker	40.7
